# Dynamical beam manipulation based on 2-bit digitally-controlled coding metasurface

**DOI:** 10.1038/srep42302

**Published:** 2017-02-08

**Authors:** Cheng Huang, Bo Sun, Wenbo Pan, Jianhua Cui, Xiaoyu Wu, Xiangang Luo

**Affiliations:** 1 State Key Laboratory of Optical Technologies on Nano-Fabrication and Micro-Engineering, Institute of Optics and Electronics, Chinese Academy of Science, P. O. Box 350, Chengdu 610209, China

## Abstract

Recently, a concept of digital metamaterials has been proposed to manipulate field distribution through proper spatial mixtures of digital metamaterial bits. Here, we present a design of 2-bit digitally-controlled coding metasurface that can effectively modulate the scattered electromagnetic wave and realize different far-field beams. Each meta-atom of this metasurface integrates two pin diodes, and by tuning their operating states, the metasurface has four phase responses of 0, π/2, π, and 3π/2, corresponding to four basic digital elements “00”, “01”, “10”, and “11”, respectively. By designing the coding sequence of the above digital element array, the reflected beam can be arbitrarily controlled. The proposed 2-bit digital metasurface has been demonstrated to possess capability of achieving beam deflection, multi-beam and beam diffusion, and the dynamical switching of these different scattering patterns is completed by a programmable electric source.

Metasurface has attracted much interest in recent years, owing to its large flexibility to modulate electromagnetic (EM) wave^1^,^2^,^3^,^4^. It is generally composed of different patterned elements on a single layer, which significantly relaxes the fabrication requirement, compared with traditional bulky metamaterials. Such metasurface has the capability of generating abrupt phase changes in the incident wavefront, and consequently it could be utilized to achieve arbitrary manipulation of wavefront through ingenious design of discontinuous interfacial phase profile. Due to the production of abrupt phase shift over the wavelength scale, metasurface overthrows the traditional physical relations among the reflected, refracted and incident beams, creating the generalized Snell’s law. So far metasurfaces based on the phase discontinuities have been realized in visible^5^,^6^, terahertz^7^,^8^ and microwave range^9^,^10^,^11^, causing a great number of intriguing applications, such as wave-front control engineering^12^,^13^, flat lens^14^,^15^, spin-orbit manipulation^16^,^17^, holographic technology^18^,^19^, and low scattering cross-sections^20^,^21^,^22^ and so on. Compared with wave-control devices using traditional geometrical optics method, using metasurfaces can significantly reduce thickness and weight, which provides a promising approach for miniaturization and system integration of optical or microwave components.

Phase modulation is one of the most important features for metasurfaces, and most of the reported metasurfaces adopt the continuous phase varying cells to achieve wave manipulation. This kind of the existing metasurfaces can be regarded as “analogously-controlled metasurfaces” by their used phase modulation method. Recently, a concept of digital metamaterials has been proposed by Giovampaola and Engheta to manipulate field distribution^23^. Just like a digital circuit, different EM responses of meta-atoms can be digitally coded, producing digital metamaterial bits. Through proper spatial mixtures of such metamaterial bits, metamaterial bytes are constructed, corresponding to different material parameters or EM functions. The same concept is also suitable for metasurface. For example, two kinds of reflective meta-atoms with 0 and π phase response can use reflection phase as the coding bit, which mimic “0” and “1” elements, respectively. When such two elements are arranged in proper distributions to construct different metasurface bytes, different EM functions could be expected. Based on this concept, digital metasurfaces have been adopted to manipulate EM radiation or scattering field by designing the coding sequences of digital meta-atoms^24^,^25^. Due to the one-to-one relationship between the coding sequence and EM function, the EM response of digital metasurface is confirmed once one coding sequence is made. In order to achieve the dynamical control of the EM functions for digital metasurfaces, lumped elements are required to be integrated into the design of the meta-atom^26^. With outside stimuli, the meta-atom could behave different EM responses which can be defined as the coding bits. By using the existing digital-controlling technology to actively tune the coding sequences, the digital metasurface could realize real-time wave manipulation. In ref. 26, 1-bit digital metasurface has been presented to construct a field-programmable reflective array antenna. Although this digital metasurface can realize multi-beam steering, its beam manipulation capability is very limited due to the use of only 1-bit digital coding.

In this article, we present a design of 2-bit digital metasurface in which each meta-atom integrates two pin diodes. By controlling the operation sates of these two pin diodes, four reflection phase responses can be obtained, which are 0, π/2, π, and 3π/2, corresponding to four basic digital elements “00”, “01”, “10”, and “11”, respectively. Therefore, the design of the coding sequences for the above four digital elements plays the great role in determining EM functionality of the 2-bit digital metasurface. Through numerical simulation and experimental verification, the designed metasurface has the ability in realizing beam deflection, multi-beam and beam diffusion. Compared with the 1-bit digital metasurface, the beam manipulation capability of this 2-bit digital metasurface is obviously enhanced.

## Results

### Structure design and its simulation results

**Figure 1:**
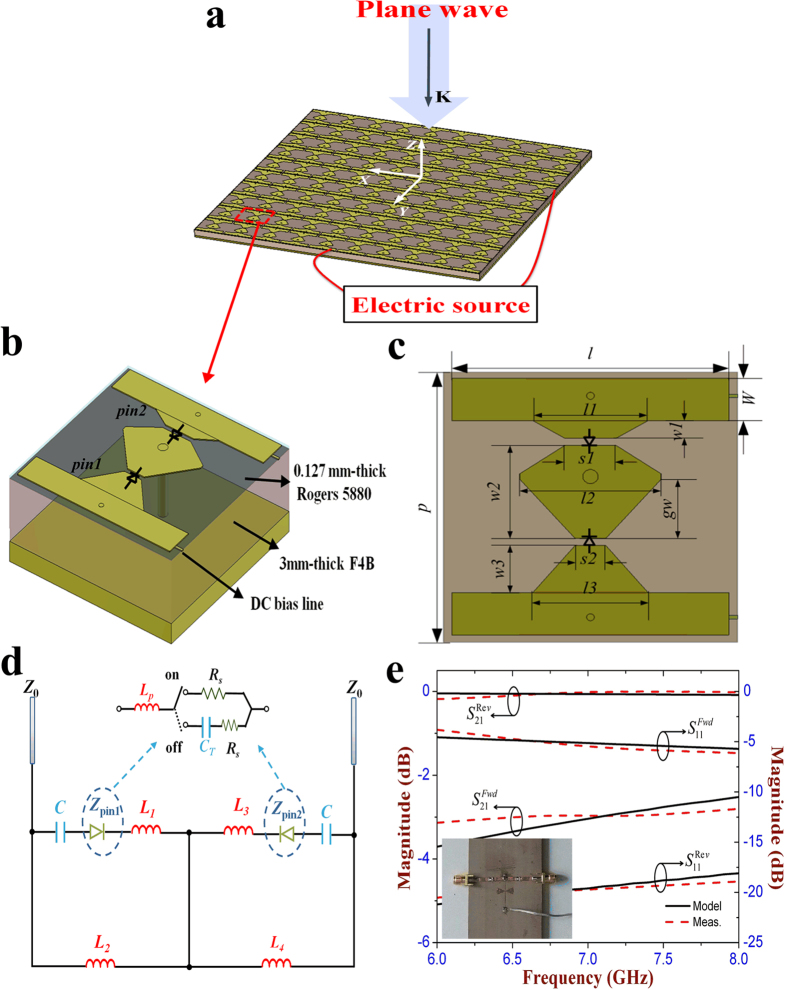
Schematic model of the designed 2-bit digital metasurface. (**a**) A 2-bit digital metasurface illuminated by a plane wave with electric field polarizing along *y* direction. The scattering pattern of this metasurface can be dynamically tuned by electric source. (**b, c**) Unit cell of the proposed 2-bit digital metasurface: (**b**) 3D-view, (**c**) Top view. (**d**) Equivalent circuit model of the designed unit cell. (**e**) Measured and modeled *S* parameters of the pin diode in the forward (*I*_*f*_ = 20 mA) and reverse (*V*_*r*_ = 5 V) bias states.

Figure 1(a) shows the schematic model of the designed 2-bit digital metasurface. Its meta-atom is able to produce four different EM responses which can be set as “00”, “01”, “10”, “11” digital bits, respectively. When designing the array of the meta-atoms in a specific coding sequence, the whole metasurface would realize the corresponding scattering pattern and it can be actively tuned by an electric source. The geometrical model of the meta-atom is depicted in Fig. 1(b). Two pairs of metallic trapezoidal patches and two rectangular patches are etched on the top layer of the Rogers 5880 dielectric substrate with thickness of 0.127 mm. A full metal sheet is placed at the bottom of the F4B dielectric substrate with thickness of 3 mm. The above two kinds of the dielectric substrates are bonded by a RO4403 film (*ε* = 3.38, *t* = 0.09 mm). Two pin diodes are inserted between gaps of two pairs of trapezoidal patches, as seen in Fig. 1(c). One pole of the pin diode is grounded through a central metallized via-hole, and the other one is connected to a bias line etched on the bottom layer of the Rogers 5880 dielectric substrate. Figure 1(d) shows the equivalent circuit model of the designed meta-atom. The metallic part can be modeled as inductor since the high frequency current flowing on it produces quasi static magnetic field. Therefore, *L*_*1*_ and *L*_*3*_are used to describe the inductance from the metallic patches on top layer, while *L*_*2*_ and *L*_*4*_ represent the inductance produced from the ground plane. The coupling capacitance from the neighboring cells is modeled as a capacitor *C*. The pin diode has two operation states, which is controlled by the bias voltage. When the pin diode works in ON state, it can be equivalent as a series circuit of parasitic inductance and resistance. If the pin diode is in OFF state, a series circuit of parasitic inductance, capacitance and resistance is used to describe it. The designed meta-atom can be considered as the termination load of the transmission line. So its effective impedance would be calculated as


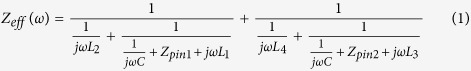


where 
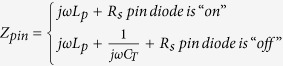
. Since the meta-atom is illuminated by a normal plane wave, the reflection coefficient can be expressed as


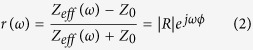


where *Z*_*0*_ = 377 Ω is the impedance of free space. Since four different impedance values of *Z*_*eff*_ can be obtained by tuning the states of these two pin diodes, the meta-atom is expected to produce four different reflection phases. The geometrical parameters indicated in Fig. 1(c) is set as follows: *l* = 10.8 mm, *w* = 1.8 mm, *l1* = *l3* = 5.2 mm, *l2* = 5.5 mm, *w1* = 0.75 mm, *w2* = 3.45 mm, *w3* = 2.5 mm, *s1* = 2.6 mm, *s2* = 1.6 mm, and *gw* = 2 mm. The 2-bit digital metasurface is designed to operate around 7.25 GHz. The M/A-COM Flip Chip MA4SPS502 is selected for the loaded pin diodes to control the reflection phase, and the electrical specification sheet of this pin diode only lists its parameters around 1 GHz^27^. In order to obtain the electric parameters around 7.25 GHz, the pin diode is mounted in parallel on a transmission line. As the inset of Fig. 1(e) shows, two DC block capacitors and the RF choke implemented with two san-shape structures are utilized for bias decoupling. A full two-port calibration is applied at the ends of the same transmission line without the diode. Then we can perform the measurement of S parameter for the pin diode in the forward (*I*_*f*_ = 20 mA) and reverse (*V*_*r*_ = 5 V) bias states, respectively. The electrical parameters of the diode equivalent model can be derived by fitting measured and simulated results, as seen in Fig. 1(e). For a forward bias current of 20 mA, the electric model is set as *R*_*s*_ = 3.8 Ω and *L*_*P*_ = 0.65 nH, while it is given by *C*_*T*_ = 0.085 pF, *L*_*P*_ = 0.65 nH for a reverse bias voltage of 5 V. Then the reflection characteristics of the proposed meta-atom are investigated under different operation states of the pin diodes, by the commercial software of CST Microwave studio 2014. A Floquet port is used to produce a *y*-polarized wave incident onto the meta-atom and receive the reflection wave. Periodic boundary condition is set to its four sides to model infinite array.

**Figure 2:**
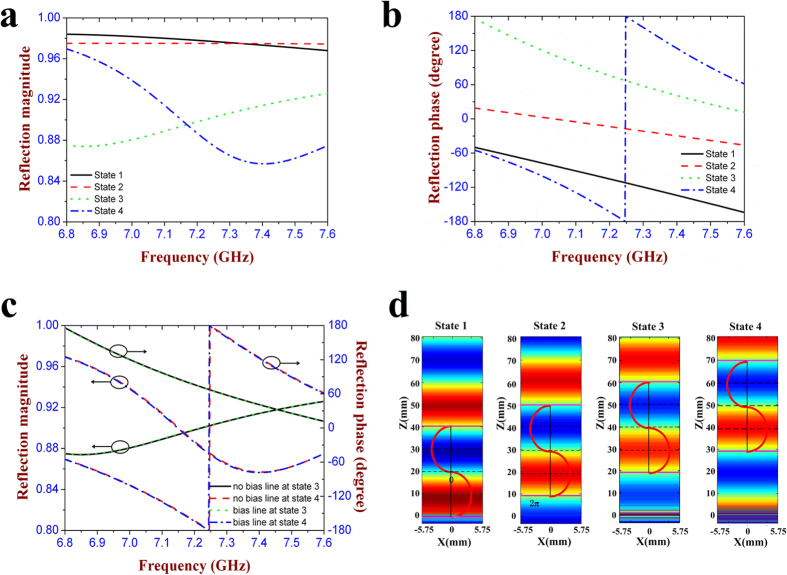
Simulated reflection characteristics of the unit cell for the proposed 2-bit digital metasurface under four different operation states of the pin diodes. (**a**) Reflection magnitude. (**b**) Reflection phase. State 1: both two pin diodes are in OFF state. State 2: both two pin diodes are in ON state. State 3: the pin diode 1 is in OFF state, while the pin diode 2 in ON state. State 4: the pin diode 1 and pin diode 2 are set to be in ON and OFF state, respectively. (**c**) Comparison of the reflection characteristics of the unit cell with and without the bias lines at state 3 and state 4. (**d**) Simulated electric-field distributions of reflection waves at each state.

**Figure 3:**
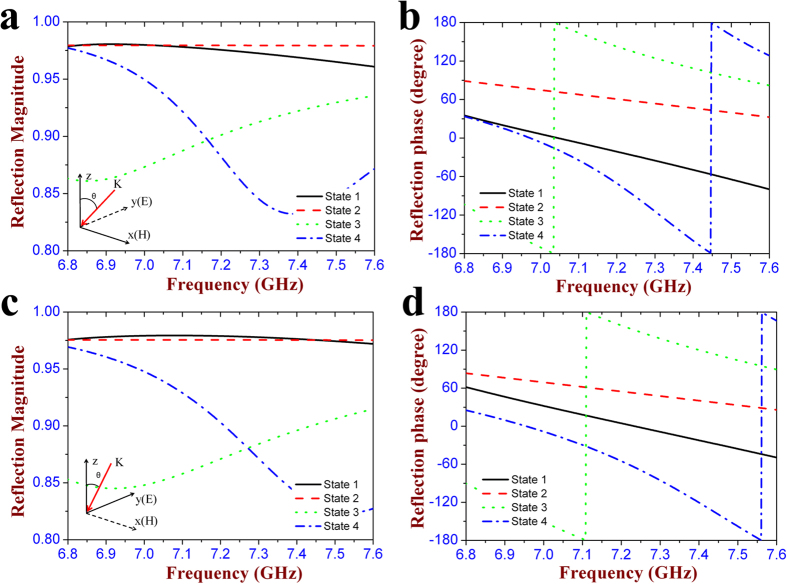
Simulation results of the designed 2-bit digital metasurface under oblique illumination. (**a,b**) Reflection magnitude and phase at the oblique incident angle of 30° in *yoz* plane. (**c,d**) Reflection magnitude and phase at the oblique incident angle of 30° in *xoz* plane.

Figure 2(a) and (b) show the reflection magnitude and phase at each state, respectively. It is seen that most of the incident wave is reflected by this meta-atom with the reflection magnitude larger than 0.85 at all the four states between 6.8 GHz and 7.6 GHz. The reflection phase is obviously different at each state, and the phase differences between neighboring states are located in the range of (90°−10°, 90° + 10°) around 7.25 GHz. That means the proposed metasurface operating from state 1 to state 4 can produce the gradient reflection phase distribution which is *θ, θ* + 90°, *θ* + 180°, and *θ* + 270°, respectively. Therefore, the cell configuration at each state can be considered as a basic digital element, and four types of cell configurations produce 2 bits, mimicking the “00” (state 1), “01” (state 2), “10” (state 3), “11” (state 4) elements, respectively. In addition, the effect of the two bias lines on the reflection performance of the meta-atom is analyzed in Fig. 2(c). As it shows, the reflection curves are almost overlapped at the state 3 and state 4 between the meta-atom with and without the bias lines, which means that the introduction of the bias lines has no influence on the EM response of the designed meta-atom. Figure 2(d) shows the simulated electric field distribution of the reflection waves in *yoz* plane at all the four states. It is seen that the *y*-polarized incident wave is vertically reflected and the wavefront has obvious phase difference among four states. The reflection phase at the present state is always 90° ahead to that of the previous state, which further demonstrates that the designed meta-atom is able to achieve 2-bit phase shifting. The reflection characteristics of this meta-atom under oblique illumination is also discussed. As the inset of Fig. 3(a) shows, a plane wave with the wave-vector in *yoz* plane is impinging on the meta-atom at an incident angle of 30 degree. Most of the incident wave is reflected and its reflection magnitude exceeds 0.82 at each state. The phase difference range between the adjacent states is increased to (90°−20°, 90° + 20°) around 7.25 GHz, as seen in Fig. 3(b). When the wave-vector is rotated to *xoz* plane with the same incident angle, it is seen from Fig. 3(c) and (d) that the designed meta-atom still keeps the high reflection magnitude at all the four states, and the phase difference is located in the range of (90°−15°, 90° + 15°). The above results have fully verified that this metasurface can still perform its digital coding property under the oblique illumination of 30 degree.

**Figure 4:**
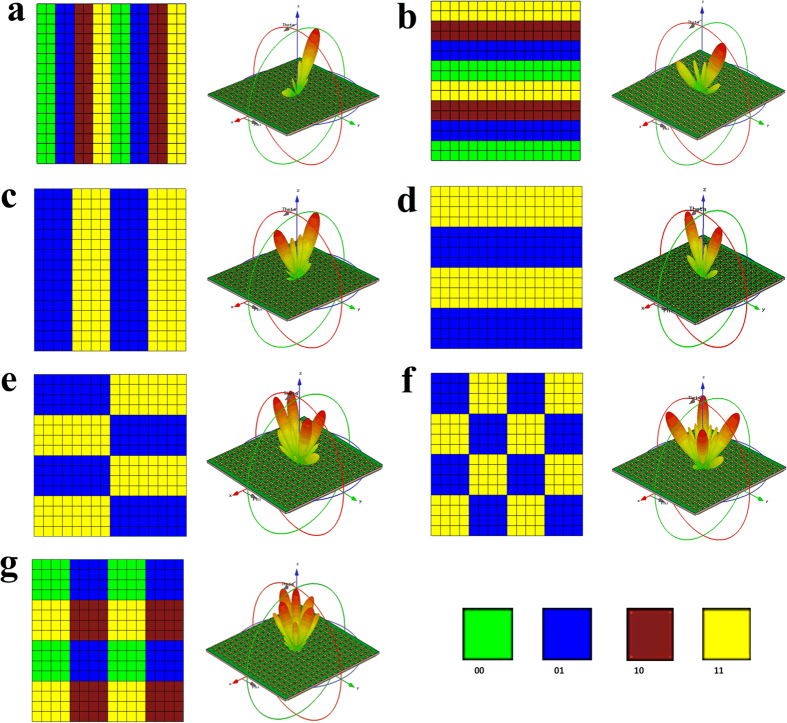
Simulated far field patterns of the 2-bit digital metasurface with different coding sequences at 7.25 GHz. (**a,b**) 3D-scattering patterns of the 2-bit digital metasurface with the coding sequence “00”, “01”, “10”, “11”… along *x* and *y* direction, respectively. The *y*-polarized reflection wave is deflected to the other direction. (**c,d**) 3D-scattering patterns of the 2-bit digital metasurface with the coding sequence “01”, “11”…along *x* and *y* direction, respectively. The *y*-polarized reflection wave is scattered into two main directions. (**e,f**) 3D-scattering patterns of the 2-bit digital metasurface with the different lattice sizes of 4 × 4 units and 4 × 8 units in the chessboard distribtuion, respectively. The *y*-polarized reflection wave is scattered into four main directions. (**g**) 3D-scattering patterns of the 2-bit digital metasurface with the coding sequence “00”, “01”/“11”, “10”. The *y*-polarized reflection wave is scattered into numerous directions, producing the beam diffusion.

The 2-bit digital metasurface composed of 16 × 16 elements is designed to investigate its beam manipulation performance. Each unit cell can be independently tuned, and different coding sequences for the full model would result in the different backward scattered fields. The whole numerical simulations are completed by using CST Microwave Studio 2014. A *y*-polarized plane wave is normally incident onto the designed 2-bit metasurface with “open (add space)” boundary condition. The backward scattered patterns under different coding sequences are shown in Fig. 4. The proposed 2-bit metasurface is able to achieve beam deflection, beam splitting and beam diffusion, respectively. For example, under the periodic coding sequences of “00”, “01”, “10”, “11”… along *x* or *y* direction, the designed metasurface makes the normally incident wave deflected to (*φ* = 180°, *θ* = 25.5°) in *xoz* plane or (*φ* = 90°, *θ* = 25.5°) in *yoz* plane, as seen in Fig. 4(a) and (b). When only two kinds of the digital elements of “01” and “11” are adopted and periodically distributed along *x* or *y* direction, the reflected wave is split into two symmetrically oriented directions in *xoz* or *yoz* plane, as shown in Fig. 4(c) and (d). If placing the above two digital elements in the chessboard distribution, the metasurface can further make the reflected wave scattered into four main beams. The beam directions of these four main lobes could be controlled by changing the lattice sizes of this digital metasurface. As Fig. 4(e) shows, the lattice size of 4 × 8 units is utilized to construct the chessboard-shape metasurface that makes the four main lobes redirected to (27°, 69°), (27°, 111°), (27°, 249°), and (27°, 291°), respectively. When the lattice of the chessboard is changed to 4 × 4 units, the four main beams would be produced in the four quadrants, *φ* = 45°, 135°, 225° and 315°, respectively, as seen in Fig. 4(f). In order to redistribute the reflected energy in more directions, all the four digital elements are utilized with a periodic “00” “01”/“11” “10” distribution. It is seen in Fig. 4(g) that the reflected wave is split into numerous main beams, thereby creating EM diffusion. Compared with the mirror reflection of the metallic flat plate with the same dimension, the production of the EM diffusion can effectively reduce radar cross section (RCS) of this 2-bit digital metasurface.

### Experimental results

**Figure 5:**
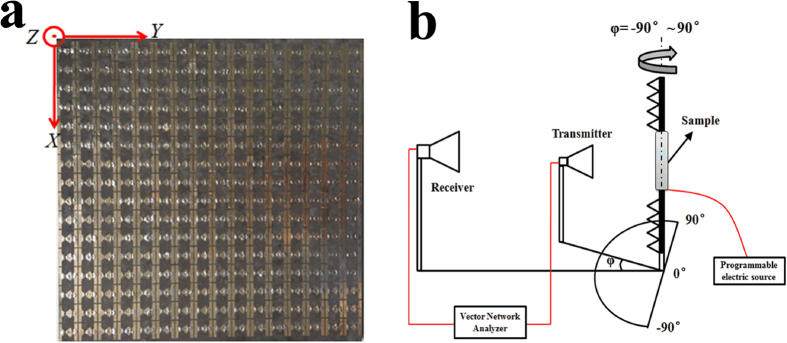
The measurement of the fabricated 2-bit digital metasurface (**a**) Photography of the fabricated metasurface and (**b**) its measurement setup for the scattering patterns.

**Figure 6:**
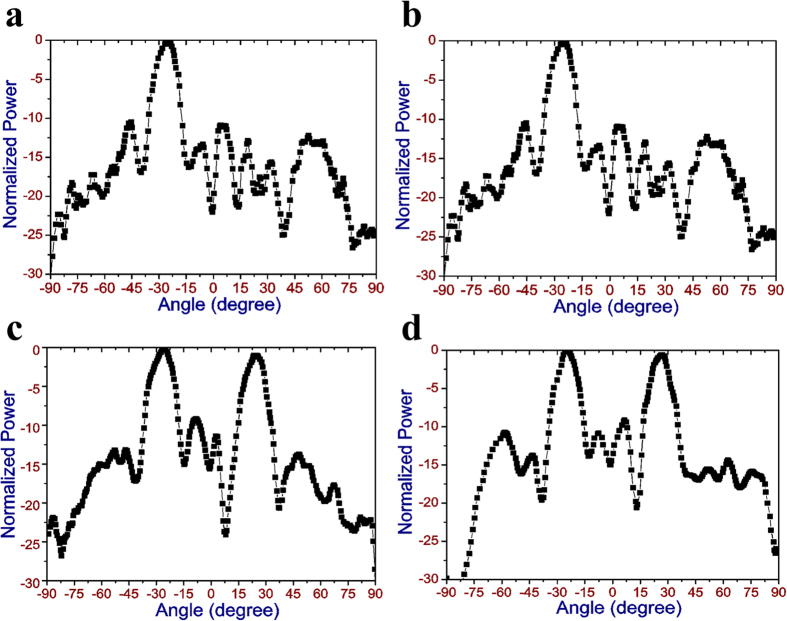
Measured far field patterns of the fabricated digital metasurface at 7.25 GHz. (**a,b**) Measured beam deflection performance of the metasurface with the coding sequence “00”, “01”, “10”, “11”… along *x* and *y* direction, respectively. (**c,d**) Two-beam splitting performance of the metasurface with the coding sequence “01”, “11”…along *x* and *y* direction, respectively.

Compared with a 1-bit digital metasurface, the proposed 2-bit digital metasurface has greater flexibility in manipulating the scattering wave for wider applications. When placing an exciting source, e.g. horn antenna in front of this metasurface, it can be used as a reflectrarray antenna for single-beam steering or producing multi-beam. In addition, it can be regarded as a low-RCS material for stealth application. Through tuning the coding sequences, the role of this metasurface can be dynamically switched among the various EM functions. To verify the simulation results of this 2-bit digital metasurface, we fabricated a sample composed of 16 × 16 units by printed circuit board technology, as seen in Fig. 5(a). A multi-way electric source is adopted to control the operation states of the pin diodes loaded on each unit of the sample. The measurement of the far field pattern is carried out in the anechoic chamber, and the schematic diagram for the measurement setup is illustrated in Fig. 5(b). Two linearly-polarized horn antennas connected to a vector network analyzer (R&S ZVA 40) are set as transmitter and receiver, respectively. The sample and the transmitting horn antenna placed in front of it is fixed on the rotation equipment. When moving the rotation equipment, both the sample and transmitting horn antenna will rotate together, so that the sample can be always illuminated by the normal incident wave. The receiving horn antenna is utilized to capture EM wave reflected by the sample for the (−90° to + 90°) rotating angles. Here, we use this measurement setup to verify the beam deflection and two-beam splitting characteristics of the fabricated sample. For the case of the beam deflection, the distribution of element array is set as “00, 01, 10, 11 …” along *x* direction through the bias voltages. It is seen in Fig. 6(a) that a single directive beam is produced in *xoz* plane and its scattering angle is about 25° deflecting from the normal of the sample. When the element array with the same coding sequence is distributed along *y* direction, the production of the single beam occurs in *yoz* plane, and its deflection angle is about 24°, as shown in Fig. 6(b). For the case of the beam-splitting, only two digital elements of “01” and “11” are adopted to construct the whole array, and its coding sequence of “01, 11, 01, 11 …” is distributed along one direction. Figure 6(c) and (d) show the measured results of this 2-bit metasurface with the above coding sequence along *x* and *y* direction, respectively. It is seen that the normally incident wave is scattered into two reflective beams by this metasurface. The two beams are respectively redirected to −26° and 24° in *xoz* plane for the *x*-direction digital coding, while the deflection angles are −25° and 25°, respectively, in *yoz* plane for the *y*-direction digital coding. All the above measured results agree well with the simulation ones, which verifies the credibility of the numerical simulation. By actively controlling the coding sequences of the designed metasurface, different scattering patterns can be realized, satisfying the various application fields.

## Discussion

In summary, we have presented a 2-bit digtial metasurface for dynamical modulation of the scattering pattern. In the unit design of the metasurface, two pin diodes are loaded to help the each meta-atom produce four phase responses of 0, π/2, π, and 3π/2, corresponding to four basic digital elements “00”, “01”, “10”, and “11”. Through proper spatial mixtures of these four digital elements, the designed metasurface has been demonstrated to achieve one-beam deflection, two- and four-beam splitting and beam diffusion by real-time control of the bias voltage. It is expected that the digitally-controlled coding metasurface can integrate more EM functions^28^,^29^ in itself with the increase of the digital bit, which may be developed for the multi-functional microwave and optical devices in the future.

## Additional Information

**How to cite this article**: Huang, C. *et al*. Dynamical beam manipulation based on 2-bit digitally-controlled coding metasurface. *Sci. Rep.* **7**, 42302; doi: 10.1038/srep42302 (2017).

**Publisher's note:** Springer Nature remains neutral with regard to jurisdictional claims in published maps and institutional affiliations.
